# Review of antibiotic prescriptions as part of antimicrobial stewardship programmes: results from a pilot implementation at two provincial-level hospitals in Viet Nam

**DOI:** 10.1093/jacamr/dlac144

**Published:** 2023-01-18

**Authors:** Ta Thi Dieu Ngan, Truong Anh Quan, Le Minh Quang, Vu Hai Vinh, Chau Minh Duc, Huynh Thi Nguyet, Nguyen Thi Cam Tu, Nguyen Hong Khanh, Le Ba Long, Nguyen Hong Hue, Dinh The Hung, Nguyen Duc Thanh, Nguyen Van Ve, Tran Thanh Giang, Le Thanh Tung, Truong Thanh Tuan, Thomas Kesteman, Elizabeth Dodds Ashley, Deverick J Anderson, H Rogier Van Doorn, Vu Thi Lan Huong

**Affiliations:** National Hospital for Tropical Diseases, 78 Giai Phong, Hanoi, Viet Nam; Hanoi Medical University, 1 Ton That Tung, Hanoi, Viet Nam; Hanoi Group, Oxford University Clinical Research Unit, 78 Giai Phong, Viet Nam; Viet Tiep Hospital, 1 Nha Thuong, Cat Dai, Le Chan, Hai Phong, Viet Nam; Viet Tiep Hospital, 1 Nha Thuong, Cat Dai, Le Chan, Hai Phong, Viet Nam; Dong Thap Hospital, 144 Mai Van Khai, My Tan, Cao Lanh City, Đong Thap; Dong Thap Hospital, 144 Mai Van Khai, My Tan, Cao Lanh City, Đong Thap; Hanoi Group, Oxford University Clinical Research Unit, 78 Giai Phong, Viet Nam; Hanoi Group, Oxford University Clinical Research Unit, 78 Giai Phong, Viet Nam; Viet Tiep Hospital, 1 Nha Thuong, Cat Dai, Le Chan, Hai Phong, Viet Nam; Viet Tiep Hospital, 1 Nha Thuong, Cat Dai, Le Chan, Hai Phong, Viet Nam; Viet Tiep Hospital, 1 Nha Thuong, Cat Dai, Le Chan, Hai Phong, Viet Nam; Viet Tiep Hospital, 1 Nha Thuong, Cat Dai, Le Chan, Hai Phong, Viet Nam; Dong Thap Hospital, 144 Mai Van Khai, My Tan, Cao Lanh City, Đong Thap; Dong Thap Hospital, 144 Mai Van Khai, My Tan, Cao Lanh City, Đong Thap; Dong Thap Hospital, 144 Mai Van Khai, My Tan, Cao Lanh City, Đong Thap; Dong Thap Hospital, 144 Mai Van Khai, My Tan, Cao Lanh City, Đong Thap; Hanoi Group, Oxford University Clinical Research Unit, 78 Giai Phong, Viet Nam; Duke Antimicrobial Stewardship Outreach Network, Duke Center for Antimicrobial Stewardship and Infection Prevention, Duke University, Durham, NC 27710, USA; Duke Antimicrobial Stewardship Outreach Network, Duke Center for Antimicrobial Stewardship and Infection Prevention, Duke University, Durham, NC 27710, USA; Hanoi Group, Oxford University Clinical Research Unit, 78 Giai Phong, Viet Nam; Nuffield Department of Medicine, Centre for Tropical Medicine and Global Health, University of Oxford, Oxford, UK; Hanoi Group, Oxford University Clinical Research Unit, 78 Giai Phong, Viet Nam

## Abstract

**Objectives:**

To investigate the feasibility of retrospective prescription-based review and to describe the antibiotic prescribing patterns to provide information for an antimicrobial stewardship programme in Viet Nam

**Methods:**

This study was conducted in two provincial-level hospitals between February and April 2020. Reviews were done by a clinical team consisting of leaders/senior doctors of each ward to assess the optimal level (optimal/adequate/suboptimal/inadequate/not assessable) of antibiotic prescriptions. Mixed-effect logistic regression at prescription level was used to explore factors associated with optimal antibiotic use.

**Results:**

The retrospective prescription-based review was accepted by study clinical wards with varied levels of participants. One hundred and eighty-three patients (326 prescriptions) in Hospital 1 and 200 patients (344 prescriptions) in Hospital 2 were included. One hundred and nineteen of the 326 (36.5%) antibiotic prescriptions in Hospital 1 and 51/344 (14.8%) antibiotic prescriptions in Hospital 2 were determined to be optimal by the review teams. The number of adequate antibiotic prescriptions were 179/326 (54.9%) and 178 (51.7%) in Hospital 1 and Hospital 2, respectively. The optimal level was lower for surgical prophylaxis antibiotics than for empirical therapy (OR = 0.06; 95% CI 0.01–0.45), higher in prescriptions in the ICU (OR = 12.00; 95% CI 3.52–40.92), higher in definitive antibiotic therapy (OR = 48.12; 95% CI 7.17–322.57) and higher in those with an indication recorded in medical records (OR = 3.46; 95% CI 1.13–10.62).

**Conclusions:**

This study provides evidence on the feasibility of retrospective prescription-based review, with adaption to the local situation. High and varying levels of optimal antibiotic prescriptions in clinical wards in hospitals were observed in Viet Nam.

## Introduction

Antimicrobial resistance (AMR) is a global concern as it poses a great threat to public health and a burden on healthcare facilities and society.^1^ Overuse, inappropriate prescribing and extensive agriculture use are some of the drivers for microorganisms developing resistance to antibiotics, including broad-spectrum and last-resort antibiotics.^2^ Low and middle-income countries (LMICs) have increasing consumption of broad-spectrum antibiotics,^3^ which further exacerbates the emergence and spread of AMR. While new antimicrobial agents in the pipeline are expected to be effective against resistant pathogens,^4^ control and optimizing the use of existing antibiotics plays a pivotal role in combating antibiotic resistance.^5^

Antimicrobial stewardship (AMS) programmes have been promoted as one of the responses to the increasing use of antibiotics, with the aim to optimize antimicrobial use, improve patient outcomes, reduce AMR and healthcare associated infections.^6,7^ Core elements of AMS programmes include: (1) leadership and commitment; (2) accountability and responsibilities; (3) AMS actions; (4) education and training; (5) monitoring and surveillance; and (6) reporting and feedback.^7^ Until now, AMS programmes have demonstrated their beneficial clinical and economic impact.^8–11^

In Viet Nam, AMS programmes have been initiated in 47% of 655 hospitals^12^ since the issuance of the Guideline for Implementation of AMS Programmes in Hospitals (Decision 772) in March 2016, by the Ministry of Health (MoH).^13^ Implementation has been at a slow pace, limited to guideline development and pre-prescription authorization, and affected by the COVID-19 pandemic.^14^ Review of antibiotic prescriptions is one of the recommended activities for AMS programmes. However, implementation of this activity is usually *ad hoc* and inadequate, as shown by our findings from a previous qualitative study in seven hospitals in our AMR network.^15–17^ Furthermore, there is a lack of understanding on physicians’ antibiotic prescribing patterns to inform AMS programmes. Studies have been mostly conducted in community settings, and for specific medical conditions (e.g. respiratory infections, surgery) and populations (e.g. children).^18^ A point prevalence survey in 36 general hospitals in Viet Nam in 2008 showed that 1573/5104 (30.8%) antibiotic prescriptions for hospitalized patients were inappropriate.^19^ Studies conducted in other LMICs reported varying proportions of inappropriate prescribing in hospitals of between 15% and 70.3%.^20,21^ Details on how antibiotic prescriptions were assessed for optimal use were not included in many studies. Such information is valuable to inform AMS efforts to improve antibiotic use in hospital settings in LMICs.

In this study, we aimed to investigate the feasibility of retrospective prescription-based review as part of an AMS programme in clinical wards in two provincial-level hospitals to assess optimal antibiotic prescribing levels, identify areas that can be targeted in the hospital AMS programme, and provide feedback to prescribers to help improve their practices. Data from this study will also provide further understanding of antibiotic prescribing patterns and optimal levels in these settings and will be beneficial for AMS implementation in Viet Nam and other LMICs.

## Methods

### Study design and setting

This study was part of an AMS implementation programme to improve antibiotic use in two general provincial-level hospitals conducted by the Oxford University Clinical Research Unit (OUCRU) Hanoi, Viet Nam in collaboration with the Duke Antimicrobial Stewardship Outreach Network, NC, USA.^22^ The two hospitals were Viet Tiep Hospital and Dong Thap General Hospital. Viet Tiep Hospital is located in Hai Phong, a large port city (2.0 million people) in northern Viet Nam, a 2000 bed general hospital with 400 000 outpatient and 100 000 inpatient visits per year. Dong Thap General Hospital is in Cao Lanh, Dong Thap, a densely populated (494 people/km^2^) province in the Mekong Delta in southern Viet Nam with 1000 bed capacity, 710 000 outpatient and 6500 inpatient visits per year. The two study hospitals will be referred to as Hospital 1 and Hospital 2 (in no particular order) to avoid identification of hospital names in the data presented.

At the time of this study, an AMS programme was newly established and the study was conducted before the implementation of AMS interventions in the clinical wards. Four clinical wards in each hospital (one surgical ICU, one surgical ward, and two internal-medicine wards) were selected by the hospital AMS teams for participating in this study. The selection was based on the discussions in the AMS team with two selection criteria: (1) the amount of antibiotics used was greater than the average of all wards in the hospital based on pharmacy reported data; and (2) willingness of the leadership in clinical wards to participate.

### Process of prescription-based retrospective review on clinical wards

Review of prescriptions was retrospectively conducted by a team consisting of the head/vice-head of each clinical ward and senior doctors. To minimize bias inherent in self-review, an AMS doctor validated a random selection of the records reviewed after being done by the review team. Training was provided for the review teams of the study wards in reviewing prescriptions and assessing antibiotic prescriptions by the AMS team and OUCRU research team. Training on analysis, interpretation and reporting review data was also provided by OUCRU to the AMS team to improve their skills in data use and feedback.

The review team planned to retrieve and review the last 50 medical records with antibiotic treatment in February 2020 per ward. If lower than 50 medical records were retrieved in February, additional medical records were retrieved from January, March and April 2020 following the same procedure. In total, 200 medical records from four study wards per hospital were expected to be reviewed. Medical records were identified and retrieved by the administrative coordinator of the AMS team from the centralized storage unit, and were sent to the review team at each study ward. The inclusion criteria for selection were: (1) patient was admitted to the hospital in the evaluation month; (2) patient was prescribed antibiotics during hospitalization; and (3) patient was hospitalized for ≥72 h.

Review forms were used to extract and record information on demographic characteristics, clinical details, microbiology culture results and antibiotic treatment regimens. Reviewers were asked to assess each antibiotic used and indicate any of the listed specific reasons for non-optimal prescriptions identified, including inappropriate antibiotic choice, spectrum, duration, dose/frequency, or route, allergy mismatch and microbiology mismatch. They were also asked to assess the overall optimal level and guideline compliance of antibiotic prescriptions. Optimal level was evaluated as ‘optimal’, ‘adequate’, ‘suboptimal’, ‘inadequate’ and ‘not assessable’ using definitions adapted from the National Antimicrobial Prescribing Survey—Australia.^23^ ‘Optimal’ antibiotic prescription is defined as following national/locally endorsed guidelines optimally including antibiotic choice, dosage, route and duration. If endorsed guidelines are absent, ‘optimal’ is considered when the antibiotic prescription covers the likely causative or cultured pathogens and there is not a narrower-spectrum antibiotic/more appropriate choice, dosage, route or duration available, or this prescription has been reviewed and endorsed by an infectious disease doctor/clinical microbiologist. ‘Adequate’ antibiotic prescription is defined as when it is not optimal but a reasonable alternative for the likely causative or cultured pathogens (duration is less than 24 h in case of surgical prophylaxis) (See review form and assessment criteria in the Supplementary data, available at *JAC-AMR* Online). The proportion of optimal antibiotic prescriptions, and proportion of prescriptions that were compliant with treatment guidelines (national guideline or locally endorsed guideline) were identified as key indicators for tracking of antibiotic use over time as part of the AMS programme.

For the purpose of mixed-effects analysis, we used two terms: optimal (‘optimal’) versus non-optimal (remaining categories). Below we presented data from the review before the implementation of AMS interventions.

### Data analysis

Data were analysed using R version 3.6.3. Continuous data were presented as median (IQR) and categorical variables were presented as count (percentage). To explore factors influencing the optimal level of antibiotic prescriptions, mixed-effects logistic regression was applied with the support of *lme4* package in R. As one patient could be prescribed multiple antibiotics, we included patient as a random effect. To explore fixed effects, we included the following variables: patient’s demographics, clinical characteristics and antibiotic prescription characteristics. Details for each fixed effect are presented in Table S1. Univariate mixed-effects analysis was first performed to assess the independent effect of each covariate. Variables were retained for multivariable analysis if the likelihood-ratio test indicated a *P* value ≤0.05 against the null model^24^ or described as having an impact on the outcome in previous studies. The fixed effect parameters were reported as OR and 95% CI.

### Ethics

The study protocol was approved by the Oxford University Tropical Research Ethics Committee (OxTREC Reference 526-19), and the Ethics Committee of the National Hospital for Tropical Diseases (08/HĐĐĐ-NĐTƯ 31 May 2019). The conduct of this study conformed to the principles embodied in the Declaration of Helsinki.

## Results

### Feasibility of prescription-based retrospective review of antibiotic prescriptions

We observed variation in the levels of participation depending on the types of wards; more active participation by doctors in non-surgical wards was observed than by those in surgical wards. Senior doctors were assigned by ward heads to complete the review form when they were not seeing patients. The review teams required between 2 weeks and 1 month to complete one review round; more time was required for the surgical wards due to lack of staff time and a lower level of participation. The AMS team reminded the surgeons and engaged the surgical leads in frequent communication to maintain awareness and commitment. The AMS coordinator received the completed review forms and summarized data to the hospital AMS team and clinical wards in their monthly ward meetings. Hospitals planned to conduct this prescription-based review quarterly following the overall 1 year AMS implementation plan. However, based on the actual performance, a quarterly review was too intensive; a semi-annual review would be more feasible.

### Patient characteristics

In Hospital 1, one medical ward retrieved 33 medical records from January to March 2020. The other three wards retrieved 50 medical records from February 2020. In Hospital 2, 50 medical records were retrieved in four wards, mostly from February 2020. Details on the number of medical records per ward per month are presented in Table S2.

A total of 383 patients were included, 183 patients in Hospital 1 and 200 patients in Hospital 2 (Table 1). The most common diagnoses were injury (trauma) and disease of respiratory or digestive systems in both hospitals. In Hospital 1 and Hospital 2, 70/183 (38.3%) and 85/200 (42.5%) patients underwent surgery, respectively. Among patients who had microbiology culture orders, only 20/44 (45.5%) in Hospital 1 and 12/48 (25.0%) patients in Hospital 2 had culture performed before initiation of the antibiotic course. Culture positivity rate was 24.5% and 10.5% in Hospital 1 (53 samples tested) and Hospital 2 (57 tested), respectively (Table S3). Gram-negative bacteria were the most frequently isolated pathogens.

**Table 1.** Patient characteristics included in the study

Characteristics	Hospital 1 (*N* = 183)	Hospital 2 (*N* = 200)
Age (years), median (IQR)	55 (37.0–70.5)	39 (99.8–62.0)
Sex (male), *n* (%)	117 (63.9)	122 (61.0)
Comorbidity, *n* (%)^a^	101 (55.2)	56 (28.0)
Source of admission, *n* (%)		
Transferred from other health facility	45 (24.6)	60 (30.0)
Community	138 (75.4)	140 (70.0)
Patients with antibiotic use prior to admission, *n* (%)	8 (4.4)	9 (4.5)
Discharge diagnosis, *n* (%)		
Injury, poisoning and certain other consequences of external causes	65 (35.5)	49 (24.5)
Diseases of the respiratory system	30 (16.4)	54 (27.0)
Diseases of the digestive system	30 (16.4)	22 (11.0)
Certain infectious and parasitic diseases	11 (6.0)	41 (20.5)
Diseases of the genitourinary system	10 (5.5)	16 (8.0)
Diseases of the circulatory system	17 (9.3)	1 (0.5%
Neoplasms	9 (4.9)	2 (1.0)
Other	11 (6.0)	15 (7.5)
Length of hospitalization (days), median (IQR)	8 (5.0–13.0)	6 (5.0–8.0)
Patients with surgery, *n* (%)	70 (38.3)	85 (42.5)
Orthopaedic	41 (58.6)	38 (44.7)
Gastrointestinal	8 (11.4)	15 (17.6)
Urinary	1 (1.4)	12 (14.1)
Other	20 (28.6)	20 (23.5)
Patients with microbiology culture performed, *n* (%)	44 (24.0)	48 (24.0)
Culture before antibiotic initiation	20 (45.5)	12 (25.0)
Microbiology culture sample, *n*	53	57
Blood, *n* (%)	22 (41.5)	15 (26.3)
Sputum, *n* (%)	17 (32.1)	26 (45.6)
Pus, *n* (%)	2 (3.8)	7 (12.3)
Urine, *n* (%)	7 (13.2)	4 (7.0)
Abdominal fluid, *n* (%)	2 (3.8)	3 (5.3)
CSF, *n* (%)	0 (0.0)	1 (1.8)
Stool, *n* (%)	3 (5.7)	0 (0.0)
Ulcer, *n* (%)	0 (0.0)	1 (1.8)

a Common comorbidity: hypertension, diabetes type 2, dyslipidaemia, gastrointestinal disease (gastroesophageal reflux disease, gastrointestinal haemorrhage), cardiovascular disease (ischaemia, heart failure, heart valve disease) and hepatitis.

### Antibiotic prescribing patterns

A total of 670 antibiotic prescriptions were reviewed (326 prescriptions in Hospital 1 and 344 prescriptions in Hospital 2). Cephalosporins were the most common antibiotics used in both hospitals: 135/326 (41.4%) and 226/344 (65.7%) prescriptions in Hospital 1 and Hospital 2, respectively (Table 2 and Figure S1).

**Table 2.** Patterns of antibiotics prescribed among studied patients in two hospitals

Characteristics	Hospital 1 (*N* = 326)	Hospital 2 (*N* = 344)
Antibiotic subclass, *n* (%)		
Cephalosporin	135 (41.4)	226 (65.7)
Fluoroquinolone	94 (28.8)	76 (22.1)
Penicillin/β-lactamase inhibitor	57 (17.5)	13 (3.8)
Carbapenem	11 (3.4)	5 (1.5)
Other	29 (9.0)	24 (7.0)
Administration route, *n* (%)		
IV	310 (95.1)	232 (67.4)
Oral	16 (5.9)	112 (32.6)
Day of treatment, median (IQR)	6 (4.0–10.0)	5 (5.0–7.0)
Antibiotic indication documented, *n* (%)	259 (79.4)	230 (66.9)
Reviewed date/stop date documented, *n* (%)	216 (66.3)	216 (62.8)
Restricted antibiotics, *n* (%)	14 (4.3)	3 (0.9)
Approval obtained	5 (35.7)	2 (66.7)
Surgical antibiotic prophylaxis, *n* (%)^a^	26 (8.0)	67 (19.5)
Surgical antibiotic prophylaxis >24 h^b^	10 (38.5)	3 (4.5)
Postoperative antibiotic prophylaxis^c^	11 (42.3)	61 (91.0)
Third-generation cephalosporin	12 (46.2)	25 (37.3)
Second-generation cephalosporin	0 (0.0)	29 (43.3)
Fluoroquinolone	9 (34.6)	10 (14.9)
Penicillin/β-lactamase inhibitor	4 (15.4)	2 (3.0)
Other	1 (3.8)	1 (1.5)
Treatment classification, *n* (%)		
Empirical therapy	259 (79.4)	263 (76.5)
Definitive therapy with known pathogens	31 (9.5)	10 (2.9)
Antibiotic prophylaxis	26 (8.0)	67 (19.5)
Assessment of each prescription, *n* (%)		
Spectrum too broad or too narrow	2 (0.6)	52 (15.1)
Inappropriate route	1 (0.3)	4 (1.2)
Inappropriate dose/frequency	2 (0.6)	43 (12.5)
Inappropriate duration	1 (0.3)	78 (22.7)
Antibiotic not required	4 (1.2)	5 (1.5)
Mismatch (microbiology/allergy)	3 (0.9)	0 (0.0)
Guideline compliance, *n* (%)		
Compliance (national/local endorsed guideline)	218 (66.9)	247 (71.8)
Definitive therapy with known pathogen	15 (4.6)	3 (0.9)
Non-compliance	11 (3.4)	87 (25.3)
No guideline available	38 (11.7)	3 (0.9)
Not assessable	26 (8.0)	4 (1.2)
Optimal level, *n* (%)^d^		
Optimal	119 (36.5)	51 (14.8)
Adequate	179 (54.9)	178 (51.7)
Suboptimal	10 (3.1)	39 (11.3)
Inadequate	1 (0.3)	73 (21.2)
Not assessable	4 (1.2)	2 (58.1)

a Denominators for this category were 26 (Hospital 1) and 67 (Hospital 2).

b Antibiotic was administered before the surgery.

c Antibiotic was administered after the surgery and with duration >24 h.

d ‘Optimal’ prescription is defined as following national/locally endorsed guidelines optimally including antibiotic choice, dosage, route and duration. If endorsed guidelines are absent, ‘optimal’ prescription is considered when antibiotic prescription covers the likely causative or cultured pathogens and there is not a narrower-spectrum antibiotic/more appropriate choice, dosage, route or duration available or this prescription has been reviewed and endorsed by an infectious disease doctor/clinical microbiologist.

Regarding surgical antibiotic prophylaxis, the most common antibiotics used were third-generation cephalosporins (12/26; 46.2%) and fluoroquinolones (9/26; 34.6%) in Hospital 1, and third-generation (25/67; 37.3%) and second-generation cephalosporins (29/67; 43.3%) in Hospital 2 (Table 2). In Hospital 1, 10/26 (38.5%) prescriptions were given for more than 24 h and in Hospital 23/67 (4.5%), with a median duration of 8.0 (IQR: 5.5–9.0) and 4.0 (IQR: 4.0–5.0) days, respectively. The number of antibiotic prescriptions identified as postoperative prophylaxis was 11/26 (42.3%) in Hospital 1 and 61/67 (91.0%) in Hospital 2, with the median of duration of 7.0 (IQR: 6.0–10.0) and 5.0 (IQR: 4.0–5.0)  days, respectively.

Most of the antibiotic regimens were classified as empirical therapy: 259/326 (79.4%) in Hospital 1; and 263/344 (76.5%) in Hospital 2. Specifically, the review team in Hospital 2 identified 52/344 (15.1%) with inappropriate spectrum (too narrow or broad), 43/344 (12.5%) with inappropriate dose or frequency, and 78/344 (22.7%) with inappropriate duration. In contrast, only 2/326 (0.6%) with inappropriate spectrum and inappropriate dose/frequency, and 1/326 (0.3%) with inappropriate duration were identified in Hospital 1 (Table 2).

Among 670 antibiotic prescriptions, 218/326 (66.9%) and 247/344 (71.8%) were compliant with the national/local endorsed treatment guidelines in Hospital 1 and Hospital 2, respectively. Thirty-eight of 326 (11.7%) antibiotic prescriptions and 26/326 (8.0%) in Hospital 1 compared with 3/344 (0.9%) and 4/344 (1.2%) in Hospital 2 were categorized as ‘no guideline available’ or ‘not assessable’, respectively. In Hospital 1, 22/26 (84.6%) ‘no guideline available’ prescriptions were in a surgical ward. Regarding optimal level, 119/326 (36.5%) antibiotic prescriptions in Hospital 1 and 51/344 (14.8%) antibiotic prescriptions in Hospital 2 were determined to be optimal by the review teams. The number of adequate antibiotic prescriptions were 179/326 (54.9%) and 178 (51.7%) in Hospital 1 and Hospital 2, respectively (Table 2).

We separately explored antibiotic prescribing practices in community-acquired pneumonia (CAP, *n* = 80) and urinary tract infections (UTI, *n* = 24) (Tables S4 and S5). The proportions of guideline compliance for empirical treatment in CAP was higher in Hospital 2 (46/48; 95.8%) than in Hospital 1 (18/32; 56.3%).

### Determinants of optimal antibiotic prescriptions

The results of multivariate mixed-effect logistic regression are presented in Table 3. Details of likelihood-ratio test results are presented in Table S6. ICU admission, length of treatment, gastrointestinal surgery, carbapenem, definitive therapy, antibiotics used for prophylaxis, antibiotics used before admission and antibiotics used in hospital-acquired infection (HAI) were significantly associated with optimal antibiotic prescribing in the univariate analysis.

**Table 3.** Multivariate mixed-effects logistic regression between optimal prescription and demographics, clinical characteristics and antibiotic prescriptions

	Antibiotic prescription		Univariate OR (95% CI)	*P* value	Multivariate OR (95% CI)	*P* value
	Non-optimal (*N* = 466)	Optimal^a^ (*N* = 164)				
Hospital, *n* (%)^b^						
Hospital 1	179 (61.3)	113 (38.7)	Ref		Ref	
Hospital 2	287 (84.9)	51 (15.1)	0.18 (0.08–0.44)	**<0**.**001**	0.10 (0.03–0.35)	**<0**.**001**
Demographics, *n* (%)						
Sex						
Female	176 (81.9)	39 (18.1)	Ref		Ref	
Male	290 (69.9)	125 (30.1)	2.56 (0.99–6.65)	0.053	1.60 (0.54–4.71)	0.39
Clinical characteristics						
Antibiotic use before admission, *n* (%)						
Yes	21 (55.3)	17 (44.7%	Ref		Ref	
No	358 (79.2)	94 (20.8%	0.17 (0.03–0.99)	**0**.**049**	0.34 (0.04–2.71)	0.31
Don’t know	87 (62.1)	53 (37.9)	1.12 (0.17–7.45)	0.91	2.93 (0.28–30.5)	0.37
Source of admission, *n* (%)						
Hospital	131 (74.4)	45 (25.6)	Ref		Ref	
Community	335 (73.9)	119 (26.1)	0.63 (0.24–1.66)	0.35	0.84 (0.27–2.65)	0.76
ICU admission, *n* (%)						
No	274 (88.1)	37 (11.9)	Ref		Ref	
Yes	192 (60.2)	127 (39.8)	12.10 (4.63–31.59)	**<0**.**001**	12.00 (3.52–40.92)	**<0**.**001**
Length of treatment (per 5 days), median (IQR)	1.0 (0.8–1.4)	1.2 (0.8–1.8)	1.75 (1.14–2.67)	**0**.**0098**	1.51 (0.90–2.52)	0.12
Antibiotic prescriptions, *n* (%)						
Antibiotic subgroups						
Penicillin/β-lactamase inhibitor	37 (61.7)	23 (38.3)	Ref		Ref	
Second-generation cephalosporin	52 (81.2)	12 (18.8)	1.43 (0.31–6.69)	0.65	2.67(0.43–12.68)	0.29
Third-generation cephalosporin	212 (80.0)	53 (20.0)	0.70 (0.23–2.10)	0.52	1.32 (0.37–4.68)	0.67
Fourth-generation cephalosporin	12 (70.6)	5 (29.4)	1.26 (0.20–7.81)	0.81	0.32 (0.02–5.01)	0.42
Carbapenem	5 (35.7)	9 (64.3)	7.10 (1.15–43.57)	**0**.**035**	3.05 (0.36–26.25)	0.31
Fluoroquinolone	125 (77.6)	36 (22.4)	0.59 (0.20–1.78)	0.35	1.04 (0.29–3.72)	0.95
Other	23 (46.9)	26 (53.1)	5.64 (1.52–20.93)	**0**.**0097**	1.33 (0.25–6.92)	0.74
Treatment classification						
Empirical therapy	375 (74.9)	126 (25.1)	Ref		Ref	
Definitive therapy with known pathogens	4 (10.5)	34 (89.5)	57.34 (11.74–279.98)	**<0**.**001**	48.12 (7.17–322.57)	**<0**.**001**
Surgical antibiotic prophylaxis	87 (95.6)	4 (4.4)	0.11 (0.02–0.67)	**0**.**017**	0.06 (0.01–0.45)	**0**.**006**
Antibiotic review after 48–72 h						
No	204 (65.8)	106 (34.2)	Ref		Ref	
Yes	262 (81.9)	58 (18.1)	0.39 (0.17–0.89)	**0**.**030**	0.63 (0.22–1.77)	0.38
Antibiotic indication documented						
No	131 (80.3)	32 (19.6)	Ref		Ref	
Yes	335 (71.7)	132 (28.3)	1.62 (0.74–3.58)	0.23	3.46 (1.13–10.62)	**0**.**030**
Antibiotic used in HAI						
No	450 (76.5)	138 (23.5)	Ref		Ref	
Yes	16 (38.1)	26 (61.9)	10.18 (2.53–40.96)	**0**.**0010**	0.95 (0.18–4.98)	0.95
Postoperative antibiotics						
No	369 (71.4)	148 (28.6)	Ref		Ref	
Yes	97 (85.8)	16 (14.2)	0.40 (0.14–1.17)	0.094	0.42 (0.11–1.55)	0.19

Multivariate mixed-effects logistic regression model included patients as random effect; fixed effects: hospital, sex, clinical characteristics (ICU admission, length of treatment, surgical type) and antibiotic prescriptions (antibiotic subgroups, treatment classification, antibiotic review after 48–72 h, antibiotic use in HAI, and postoperative antibiotics). The analysis excluded missing values. Bold type indicates statistical significance.

a ‘Optimal’ prescription is defined as following national/locally endorsed guidelines optimally including antibiotic choice, dosage, route and duration. If endorsed guidelines are absent, ‘optimal’ prescription is considered when antibiotic prescription covers the likely causative or cultured pathogens and there is not a narrower-spectrum antibiotic/more appropriate choice, dosage, route or duration available or this prescription has been reviewed and endorsed by an infectious disease doctor/clinical microbiologist.

b All factors were adjusted for hospital for univariate mixed-effects model.

However, in multivariate analysis, only ICU admission, antibiotic indication documented, and treatment classification (definitive therapy and antibiotic prophylaxis) remained statistically significant. Antibiotics used in patients admitted to the ICU were more likely to be optimal (OR = 12.00; 95% CI 3.52–40.92) compared with non-ICU patients. Antibiotics used as definitive therapy in patients with known pathogens were also more likely to be optimally prescribed than in empirical therapy (OR = 48.12; 95% CI 7.17–322.57). Antibiotics prescribed as prophylaxis were more likely to be non-optimal than empirical therapy (OR = 0.06; 95% CI 0.01–0.45). Finally, documentation of antibiotic indication in medical records was associated with antibiotic prescriptions being more optimal (OR = 3.46; 95% CI 1.13–10.62).

## Discussion

In this paper, we described the process of conducting a retrospective prescription-based review as part of AMS implementation in hospital settings in Viet Nam. Despite recommendations in the national guideline for monitoring the quality of antibiotic prescriptions in hospitals, implementation remains challenging.^17^ Innovative strategies are required to integrate AMS activities into the routine hospital work to achieve acceptability and sustainability in the long term.^22^

Two audit interventions are recommended in the AMS practical toolkit issued by the WHO in 2019.^7^ The first and prioritized one is prospective audit and feedback (PAF); the second one is a retrospective prescription-based review with feedback. PAF provides prescribers with recommendations for each patient in real time and hence could be beneficial for patients while they are still in hospital. This approach might not be feasible for resource-limited hospitals where there are insufficient AMS staff capacity and information technology resources to perform PAF efficiently and effectively. The retrospective prescription-based review with feedback could be feasible because it requires fewer resources while still providing opportunities to improve prescribing practices of physicians, albeit not in real time. It can be done by self-revision by prescribers, which furthermore facilitates their autonomy and awareness of their antibiotic use.^25^ In our study, the review form, as well as assessment criteria, was developed with optimization to be less time-consuming yet adequate data to inform and evaluate the AMS programme.^26,27^ The implementation was well integrated into the medicinal wards while more efforts were needed in surgical wards. Identifying strategies to increase the involvement of surgeons in reviewing their own prescriptions and in AMS activities is important for the overall success of AMS programmes. Engaging surgical leads in integrated AMS and infection management discussions could potentially increase their involvement in the process.^28^

We observed high levels of non-optimal antibiotic prescribing in the study wards in two provincial-level hospitals (58.3% and 85.2% in Hospital 1 and Hospital 2, respectively). This level is comparable to those reported from Pakistan (70.3%; 2017),^21^ but much higher than those reported in other studies: Turkey (46.7%; 2007),^29^ Viet Nam (30.8%; 2008).^19^ Our data also indicated high levels of prolonged antibiotic use for surgical prophylaxis (>24 h) and use as postoperative prophylaxis, mostly in Hospital 2, with 62/67 surgical antibiotic prophylaxis prescriptions assessed as inappropriate duration, and 28/67 as inappropriate dose/frequency. As described elsewhere,^30,31^ this is possibly attributable to the doctors’ intentions to compensate for inadequate wound care and infection prevention and control procedures in the hospital, limited knowledge of spectrum of antibiotic activity and optimal timing of antibiotic prophylaxis.^32–34^ These prescribing patterns in surgical antibiotic prophylaxis do not yield any additional benefit in reducing surgical site infection but increase resistance rates and costs.^35–37^ AMS programmes in Viet Nam should specifically be tailored to prioritize the optimization of surgical antibiotic prophylaxis and integrate with infection prevention and control activities.

In Viet Nam, the MoH issued the Guideline for Antibiotic Treatment in 2015 and hospitals developed their local guidelines (last update in 2020 in two hospitals). Local treatment guidelines developed were based on MoH’s guideline, covering both internal medicine and surgery. The guideline compliance rate in our study was similar to other studies: Namibia (62.0%; 2017),^38^ Norway (60%; 2018)^39^ and Australia (65.3%; 2019),^40^ In our study, ‘no guideline available’ prescriptions were observed mostly in a surgical ward, which provides most orthopaedics and traumatology care, thus local treatment guidelines should include more guidance on surgical patient populations, especially antibiotic prophylaxis.

Our regression results showed that ICU-admitted patients were deemed to receive more optimal antibiotic therapy than non-ICU patients. This could be attributable to the fact that ICU doctors in our hospitals ordered microbiology culture for their patients more than those treating non-ICU patients (50/102; 32.9% ICU-admitted patients with culture, and 54/177; 23.4% for those not admitted to ICU). It is worth noting that AMS programmes in ICU settings have some unique consideration given the complex patients, high rate of AMR and heavy antibiotic consumption. Specific AMS interventions have been proven to be effective including de-escalation, pharmacokinetic/pharmacodynamic dose optimization, PAF and diagnosis test stewardships.^41,42^ From our findings, retrospective prescription-based review could also be a useful intervention for ICUs in LMIC settings.

Although our regression results showed that definitive therapy with known pathogens is more optimal than empirical therapy, low use of microbiology for antibiotic treatment guidance is common in hospitals in Viet Nam and other LMICs. There was only a small fraction of microbiology testing used to inform antibiotic decision-making despite a large number of test orders due to non-causative findings, non-relevant findings and a high rate of negative findings.^43^ Specifically, doctors expressed concerns about prolonged turnaround times for the microbiology cultures.^17^ Lack of laboratory infrastructure and capacity also poses a big challenge, as evident in one of our studied hospitals where blood cultures were performed manually and resistant mechanism identification (e.g. ESBL or carbapenemase) could not be performed. Lack of capacity, together with the common use of antibiotics prior to hospital admissions, might explain the low positivity rates in our study, as raised by the hospital staff in our AMS team discussions. The issue of limited resources in the microbiology laboratory setting, coupled with the scarce communication between doctors and clinical microbiologists, are the hindrance for improvement in microbiology testing practices.^22,44,45^

This study has several strengths including representativeness of hospitals in terms of: geographical regions, size and provincial-level hospitals; the involvement of doctors in reviewing their prescribing practices that could facilitate behaviour change in AMS programmes; and the feasibility for the activity to be integrated into the routine hospital work. Nevertheless, interpretations of the study data need to be made in the light of its limitations. First, small sample sizes of medical records increased the feasibility of the activity but limited the statistical power in data analysis. Second, the fact that retrospective review was performed by the clinical team at each ward could generate some bias in the indicators of optimal antibiotic prescriptions and guideline compliance. However, engagement of clinical teams in reviewing their own prescriptions could be considered as an intervention in itself because through the review process, the clinical teams would become more aware of their issues and more likely improve their practices. Lastly, the application of appropriateness categories can vary between reviewers and between studies. Although we had an AMS doctor re-evaluate the reviewed medical records to minimize the last two limitations, this was only done in one hospital because this approach depends on the staff available at each hospital and would have high cost implications for the AMS programmes.

In conclusion, retrospective prescription-based review could be integrated as part of hospital AMS implementation in LMIC settings in order to identify areas for potential antibiotic prescribing improvement. This success is linked to the fact that our programme is tailored to the local situation. Data from our prescription reviews showed wide variations in antibiotic prescribing practices in hospitals in Viet Nam. We identified parameters for improvement of antibiotic prescriptions including improving the content of hospital-specific guidelines, enhancing antibiotic use in surgery, and building microbiology laboratory capacity to support antibiotic decision-making.

## Supplementary Material

dlac144_Supplementary_Data

## Supplementary data

Figure S1, Tables S1 to S6 and the Retrospective Medical Record Review Form are available as Supplementary data at *JAC-AMR* Online.
