# Supplementary material for: Review of antibiotic prescriptions as part of antimicrobial stewardship programmes: results from a pilot implementation at two provincial-level hospitals in Viet Nam
Source: JAC Antimicrob Resist. 2023 Jan 18;5(1):dlac144. doi: 10.1093/jacamr/dlac144 (PMC9847554; doi:10.1093/jacamr/dlac144)
Supplement: dlac144_Supplementary_Data [file dlac144_supplementary_data.docx]

**SUPPLEMENTARY**

**Review of antibiotic prescriptions as part of antimicrobial stewardship programmes: results from a pilot implementation at two provincial-level hospitals in Vietnam**

**Authors:** TA Thi Dieu Ngan, TRUONG Anh Quan, LE Minh Quang, VU Hai Vinh, CHAU Minh Duc, HUYNH Thi Nguyet, NGUYEN Thi Cam Tu, NGUYEN Hong Khanh, LE Ba Long, NGUYEN Hong Hue, DINH The Hung, NGUYEN Duc Thanh, NGUYEN Van Ve, TRAN Thanh Giang, LE Thanh Tung, TRUONG Thanh Tuan, Thomas KESTEMAN, Elizabeth Dodds ASHLEY, Deverick J. ANDERSON, H Rogier van DOORN, VU Thi Lan Huong

1. Figure S1. The number of antibiotic prescriptions for both hospitals in the study
2. Table S1. Detail on fixed effects in mixed-effects logistic regression
3. Table S2. Number of medical records retrieved in each ward and month
4. Table S3. Isolated organisms in the study
5. Table S4. Antibiotic prescription in community-acquired pneumonia
6. Table S5. Antibiotic prescription in urinary tract infection
7. Table S6. Likelihood ratio test result for fixed effects
8. Retrospective Medical Record Review Form


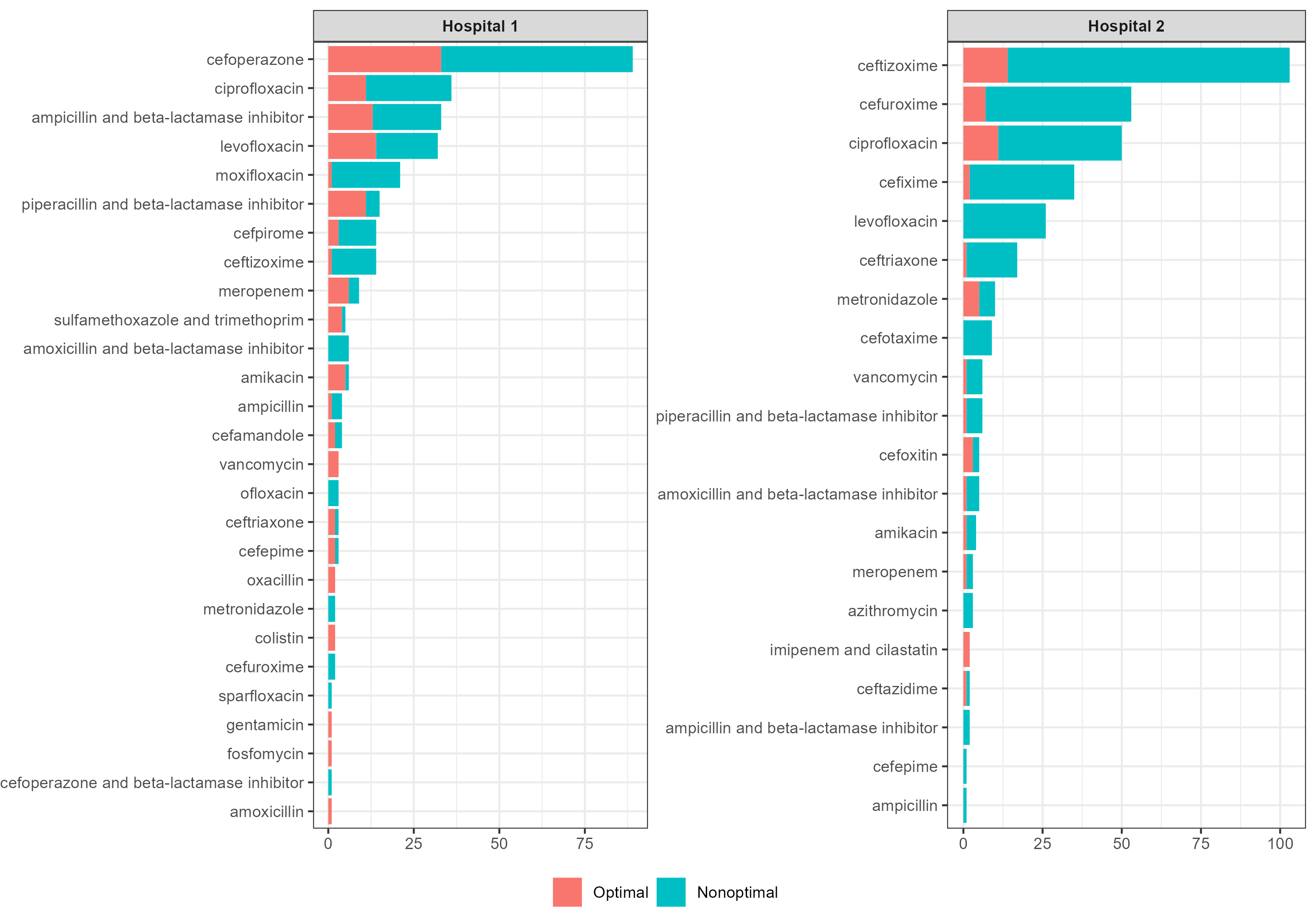


**Fig S1. The number of antibiotic prescriptions for both hospitals in the study. Antibiotic prescription was classified as optimal (“optimal”) and nonoptimal (“adequate”/ “suboptimal”/ “inadequate”) by review doctors.**

| **Table S1. Detail on fixed effects in mixed-effects logistic regression** | | |
| --- | --- | --- |
| **Fixed effects** | **Type of variables** | **Description** |
| Site | Categorical | Hospital 1/Hospital 2 |
| Patient characteristics | | |
| Sex | Categorical | No/Yes |
| Age | Continuous | Per 10 years of age |
| Clinical characteristics | | |
| Antibiotic use before admission | Categorical | No/Yes |
| Intensive care unit admission | Categorical | No/Yes |
| Length of treatment | Continuous | Per 5 days |
| Surgical type | Categorical | No surgery  Orthopedics and traumatology  Gastrointestinal  Urinary  Other |
| Comorbidity | Categorical | No/Yes |
| Source of admission | Categorical | Healthcare facilities  Home |
| Diagnosis | Categorical | Injury, poisoning and certain other consequences of external causes  Diseases of the respiratory system  Diseases of the digestive system  Certain infectious and parasitic diseases  Diseases of the circulatory system  Diseases of the genitourinary system  Other |
| Antibiotic prescription characteristics | | |
| Antibiotic subgroups | Categorical | Penicillin/β-lactamase inhibitors  2^nd^ generation cephalosporine  3^rd^ generation cephalosporine  4^th^ generation cephalosporine  Carbapenem  Fluoroquinolone  Other |
| Treatment classification | Categorical | Empirical therapy  Definitive therapy with known pathogens  Surgical antibiotic prophylaxis |
| Review of antibiotic use | Categorical | No/Yes |
| Antibiotics used in HAI | Categorical | No/Yes |
| Indication recorded in MR | Categorical | No/Yes |
| Administration route | Categorical | Oral/Parenteral |
| Prophylaxis antibiotic ≥24h | Categorical | No/Yes |
| Postoperative antibiotics | Categorical | No/Yes |

| **Table S2. Number of medical records retrieved in each ward and month** | | | | | |
| --- | --- | --- | --- | --- | --- |
|  | **Hospital 1 (N=183)** | | | **Hospital 2 (N=200)** | |
|  | **01/2020** | **02/2020** | **03/2020** | **02/2020** | **04/2020** |
| Surgical ICU | 0 | 50 | 0 | 50 | 0 |
| Surgical ward | 0 | 50 | 0 | 49 | 1 |
| Medicine ward (1) | 0 | 50 | 0 | 50 | 0 |
| Medicine ward (2) | 6 | 16 | 11 | 49 | 1 |

| **Table S3. Isolated organisms in the study** | | |
| --- | --- | --- |
|  | **Hospital 1** | **Hospital 2** |
| **Total samples** | 53 | 57 |
| **Pus** | 2 | 7 |
| *Streptococcus aureus* | 1/2 | 0/4 |
| *Escherichia coli* | 0/2 | 1/4 |
| **Sputum** | 17 | 26 |
| *Acinetobacter baumannii* | 2/17 | 0/25 |
| *Pseudomonas aeruginosa* | 3/17 | 0/25 |
| *Klebsiella pneumoniae* | 2/17 | 0/25 |
| *Streptococcus aureus* | 2/17 | 0/25 |
| **Urine** | 7 | 4 |
| *Candida* spp | 1/7 | 0/4 |
| **Abdominal fluid** | 2 | 3 |
| *Escherichia coli* | 0/2 | 2/3 |
| *Candida* spp | 1/2 | 0/3 |
| **Ulcer** | 0 | 1 |
| *Escherichia coli* | 0/0 | 1/1 |
| **Blood** | 22 | 15 |
| *Klebsiella spp* | 0/22 | 1/15 |
| Coagulase-negative staphylococci | 0/22 | 1/15 |
| *Escherichia coli* | 1/22 | 0/15 |
|  | | |

| **Table S4. Antibiotic prescription in community-acquired pneumonia** | | |
| --- | --- | --- |
|  | **Hospital 1**  **(N = 32)** | **Hospital 2**  **(N = 48)** |
| **Blood culture performed,** n (%) | 8 (25.0%) | 10 (20.8%) |
| Blood culture performed before antibiotics initiation | 4 (50.0%) | 2 (20.0%) |
| **Sputum/Endotracheal aspirate/pleural fluid Gram stain and culture,** n (%) | 10 (31.2%) | 30 (62.5%) |
| Before antibiotics initiation | 8 (80.0%) | 7 (23.3%) |
| **Virus test perform,** n (%) | 3 (9.38%) | 0 (0.0%) |
| Treat with antivirals when positive | 1 (33.33%) | 0 (0.0%) |
| **Patients having antibiotic change after culture result available,** n (%) | 3 (9.38%%) | 1 (2.08%) |
| **Patients having antibiotic stop by alternative diagnosis,** n (%) | 4 (12.5%) | - |
| **Patients having antibiotic continue by alternative diagnosis,** n (%) | 2 (6.25%) | 2 (4.17%) |
| **Patients prescribed IV antibiotics with good bioavailability,** n (%) | 6 (18.8%) | 10 (20.8%) |
| Change from IV to PO | 2 (33.3%) | 2 (20.0%) |
| **Empirical treatment compliant with guideline,** n (%) | 18 (56.3%) | 46 (95.8%) |
| **Treatment duration**, median [IQR] | 10 [6.8-11.3] | 7 [5.5 – 8.0] |
| **Planned treatment duration after discharge,** median [IQR] | 4.5 [1.3-7.0] | 5.0 [5.0-5.0] |

| **Table S5. Antibiotic prescription in urinary tract infection** | | |
| --- | --- | --- |
|  | **Hospital 1**  **(N = 14)** | **Hospital 2**  **(N = 10)** |
| **Urinalysis perform, n (%)** | 11 (78.6%) | 7 (70.0%) |
| Evidence of pyuria | 6 (54.6%) | 5 (71.4%) |
| Epithelial cell presented | 2 (28.6%) | 3 (42.9%) |
| **Urine culture, n (%)** | 6 (42.9%) | 4 (40.0%) |
| Positive result | 3 (50.0%) | 1 (25.0%) |
| Antibiotic used before culture | 6 (100.0%) | 4 (100.0%) |
| Stop empirical antibiotic after negative result of culture | 2 (33.3%) | 0 (0.0%) |
| Change antibiotic | 4 (66.7%) | 1 (25.0%) |
| **Patients prescribed IV antibiotics with good bioavailability,** n (%) | 10 (71.4%) | 8 (80.0%) |
| Change from IV to PO | 0 (0.0%) | 6 (75.0%) |
| **Guideline compliance,** n (%) | 6 (42.86%) | 10 (100%) |
| **Treatment duration,** median [IQR] | 8.0 [5.0-12.0] | 7.5 [5.3-8] |

| **Table S6. Likelihood ratio test result for fixed effects** | | |
| --- | --- | --- |
| **Fixed effects** | **P value** | **Retained in multivariate model** |
| Hospital | **<0.001** | **Yes** |
| Sex | **0.004** | **Yes** |
| Comorbidity | 1.00 | No |
| Antibiotic prior to admission | **<0.001** | **Yes** |
| Source of admission | **<0.001** | **Yes** |
| ICU admission | **<0.001** | **Yes** |
| Surgical site | **<0.001** | **Yes** |
| Diagnosis | 1.00 | **Yes** |
| Antibiotic subgroups | **<0.001** | **Yes** |
| Administration route | 1.00 | No |
| Indication recorded in MR | **<0.001** | **Yes** |
| Review of antibiotic use | **<0.001** | **Yes** |
| Antibiotic used in HAI | **<0.001** | **Yes** |
| Treatment classification | **<0.001** | **Yes** |
| Prophylaxis antibiotic ≥24hr | 1.00 | No |
| Postoperative antibiotic prophylaxis | **<0.001** | **Yes** |
| Age (per 10 years of age) | 1.00 | No |
| Length of treatment (per 5 days) | **<0.001** | **Yes** |

**Retrospective Medical Record Review Form**
